# Supplementary material for: The role of GABA in semantic memory and its neuroplasticity
Source: eLife. 2025 Jun 3;12:RP91771. doi: 10.7554/eLife.91771 (PMC12133155; doi:10.7554/eLife.91771)
Supplement: Supplementary file 1. [file elife-91771-supp1.docx]

**Supplementary information**

Justification of target site

Supplementary File 1A

Supplementary File 1B

Supplementary File 1C

Supplementary File 1D

Justification of target site selection and cTBS effects

Evidence suggests that bilateral ATL systems contribute to semantic representation (for a review, see 1). Consistent with this, our semantic task induced bilateral ATL activation (Figure 2-figure supplement 2). Thus, stimulating both left and right ATL could provide a more comprehensive understanding of cTBS effects and its GABAergic function.

Previous rTMS studies have applied inhibitory stimulation to both the left and right ATL, demonstrating that stimulation at either site significantly disrupted semantic task performance (2-4). Importantly, these studies reported no significant difference in rTMS effects between left and right ATL stimulation, suggesting that stimulating either hemisphere produces comparable effects on semantic processing. In the current study, we combined cTBS with multimodal imaging to investigate its effects on the ATL. Given our study design constraints (including the need for a control site, control task, and control stimulation) and limitations in scanning time, we selected the left ATL as the target region. This choice also aligned with the MRS voxel placement used in our previous study (5), allowing us to combine datasets and further investigate GABAergic function in the ATL. Accordingly, cTBS was applied to the peak coordinate of the left ventromedial ATL (MNI -36, -15, -30) as identified by previous fMRI studies (6, 7).

Given that TMS pulses typically penetrate 2–4 cm, we acknowledge the challenge of reaching deeper ventromedial ATL regions. However, our findings indicate that cTBS effectively modulated ATL function, as evidenced by reduced task-induced regional activity, increased ATL GABA concentrations, and poorer semantic performance, confirming that TMS pulses successfully influenced the target region. To further validate these effects, we conducted an ROI analysis centred on the ventromedial ATL (MNI -36, -15, -30), which revealed a significant reduction in ATL activity during semantic processing following ATL stimulation (t = -2.43, p = 0.014) (Figure 2-figure supplement 5). This confirms that cTBS successfully modulated ATL activity at the intended target coordinate.

Supplementary File 1A

| Session | Label | | | Ks | T | MNI x, y, z |
| --- | --- | --- | --- | --- | --- | --- |
| ATL pre-stimulation | L Fusiform Gyrus | 3278 | | | 11.08 | -33 -42 -24 |
|  | L Inferior Occipital Gyrus |  | | | 10.4 | -39 -69 -12 |
|  | L Fusiform Gyrus | |  | | 10.07 | -39 -60 -12 |
|  | L Fusiform Gyrus | |  | | 10.89 | -36 -15 -26 |
|  | R Fusiform Gyrus | | 2931 | | 10.43 | 39 -51 -15 |
|  | R Middle Occipital Gyrus | |  | | 9.55 | 45 -78 0 |
|  | L IFG (p. Triangularis) | | 1194 | | 7.48 | -51 27 18 |
|  | L IFG (p. Triangularis) | |  | | 6.72 | -45 33 6 |
|  | L IFG (p. Triangularis) | |  | | 6.54 | -36 33 0 |
|  | R IFG (p. Triangularis) | | 368 | | 6.55 | 42 21 24 |
|  | R IFG (p. Opercularis) | |  | | 6.51 | 39 12 24 |
|  | R IFG (p. Opercularis) | |  | | 5.76 | 48 30 15 |
|  | R Supplementary Motor Area | | 272 | | 4.93 | 9 12 48 |
|  | L Supplementary Motor Area | |  | | 4.76 | -3 15 48 |
|  | R MCC | |  | | 4.6 | 12 21 33 |
| ATL post-session | L Fusiform Gyrus | | 3647 | | 12 | -33 -42 -21 |
|  | R Fusiform Gyrus | |  | | 11.64 | 30 -45 -18 |
|  | L Inferior Occipital Gyrus | |  | | 11.45 | -39 -69 -12 |
|  | L IFG (p. Triangularis) | | 859 | | 9.05 | -51 27 15 |
|  | L IFG (p. Triangularis) | |  | | 7.41 | -45 23 6 |
|  | R IFG (p. Triangularis) | | 521 | | 6.33 | 42 24 24 |
|  | R IFG (p. Triangularis) | |  | | 5.56 | 48 30 15 |
|  | R Insula Lobe | |  | | 4.72 | 36 27 -6 |
|  | L Supplementary Motor Area | | 356 | | 5.55 | -3 18 48 |
|  | R Supplementary Motor Area | |  | | 5.47 | 9 12 51 |
|  | R MCC | |  | | 4.21 | 12 24 33 |
| Vertex pre-stimulation | L Fusiform Gyrus | | 2884 | | 10.76 | -36 -42 -21 |
|  | L Fusiform Gyrus | |  | | 9.69 | -39 -66 -15 |
|  | L Inferior Occipital Gyrus | |  | | 8.78 | -42 -78 -6 |
|  | R Fusiform Gyrus | | 2950 | | 10.11 | 36 -54 -18 |
|  | R Fusiform Gyrus | |  | | 9.83 | 33 -45 -18 |
|  | R Middle Occipital Gyrus | |  | | 9.61 | 45 -78 -3 |
|  | L IFG (p. Triangularis) | | 1213 | | 7.97 | -42 21 21 |
|  | L IFG (p. Triangularis) | |  | | 7.57 | -51 27 15 |
|  | L Middle Frontal Gyrus | |  | | 5.36 | -33 18 54 |
|  | R IFG (p. Triangularis) | | 302 | | 5.93 | 51 33 12 |
|  | R IFG (p. Triangularis) | |  | | 4.83 | 42 12 27 |
|  | R IFG (p. Opercularis) | |  | | 4.75 | 45 21 24 |
|  | R SMA | | 180 | | 5.15 | -3 15 51 |
|  | L Supplementary Motor Area | |  | | 4.43 | 6 24 48 |
|  | L Superior Medial Gyrus | |  | | 4.03 | -6 27 42 |
| Vertex post-session | L Fusiform Gyrus | | 3403 | | 11.59 | -36 -42 -24 |
|  | L Fusiform Gyrus | |  | | 9.65 | -39 -66 -12 |
|  | L Inferior Occipital Gyrus | |  | | 8.82 | -42 -75 -9 |
|  | R Fusiform Gyrus | | 3279 | | 9.6 | 33 -45 -18 |
|  | R Fusiform Gyrus | |  | | 9.57 | 39 -51 -15 |
|  | R Inferior Temporal Gyrus | |  | | 9.18 | 42 -69 -3 |
|  | L IFG (p. Triangularis) | | 1478 | | 7.19 | -42 21 21 |
|  | L IFG (p. Triangularis) | |  | | 7.09 | -45 30 15 |
|  | L IFG (p. Orbitalis) | |  | | 5.24 | -42 42 -6 |
|  | R IFG (p. Opercularis) | | 282 | | 6.01 | 42 12 27 |
|  | R IFG (p. Triangularis) | |  | | 5.11 | 54 33 18 |
|  | R IFG (p. Triangularis) | |  | | 4.42 | 57 27 24 |
|  | R Supplementary Motor Area | | 315 | | 4.83 | 0 18 51 |
|  | R Supplementary Motor Area | |  | | 4.4 | 12 -3 45 |
|  | L Superior Medial Gyrus | |  | | 4.03 | -9 27 60 |

Supplementary File 1A. The results of fMRI (semantic > control)

Supplementary File 1B

|  |  | Accuracy (%) | | | |  | Reaction time (s) | | | |  |
| --- | --- | --- | --- | --- | --- | --- | --- | --- | --- | --- | --- |
|  |  | PRE | | POST | |  | PRE | | POST | |  |
|  |  | mean | SD | mean | SD |  | mean | SD | mean | SD |  |
| ATL stimulation | Semantic | 82 | 8 | 82 | 6 |  | 1.99 | 0.44 | 1.97 | 0.44 | t = 0.37, p = 0.720 |
|  | Control | 97 | 5 | 98 | 2 |  | 1.71 | 0.24 | 1.53 | 0.26 | t = 3.27, p = 0.005 |
| Vertex stimulation | Semantic | 78 | 12 | 80 | 10 |  | 2.11 | 0.40 | 2.00 | 0.43 | t = 2.65, p = 0.017 |
|  | Control | 95 | 5 | 96 | 5 |  | 1.75 | 0.36 | 1.64 | 0.36 | t = 2.24, p = 0.040 |

Supplementary File 1B. The results of behavioural performance. SD represent the standard deviation.

Supplementary File 1C

| Control task | | ATL stimulation | | Vertex stimulation | |
| --- | --- | --- | --- | --- | --- |
|  |  | Pre-stimulation | Post-stimulation | Pre-stimulation | Post-stimulation |
| Accuracy | r | -0.37 | 0.293 | 0.332 | -0.149 |
|  | p | 0.192 | 0.309 | 0.246 | 0.621 |
| RT | r | -0.18 | 0.293 | -0.008 | 0.501 |
|  | p | 0.538 | 0.309 | 0.978 | 0.068 |

Supplementary File 1C. The partial correlation results between control task performance and ATL GABA levels accounting for GM volume, age, and sex.

Supplementary File 1D

|  | ATL VOI | | Vertex VOI | |
| --- | --- | --- | --- | --- |
|  | PRE | POST | PRE | POST |
| ATL stimulation | 0.50 ± 0.03 | 0.47 ± 0.03 | 0.24 ± 0.03 | 0.22 ± 0.03 |
| Vertex stimulation | 0.47 ± 0.03 | 0.49 ± 0.02 | 0.29 ± 0.03 | 0.27 ± 0.04 |

Supplementary File 1D. The summary of tissue type within MRS VOIs. Mean ± SE

**References**

1. M. A. Ralph, E. Jefferies, K. Patterson, T. T. Rogers, The neural and computational bases of semantic cognition. *Nat Rev Neurosci* **18**, 42-55 (2017).

2. M. A. Lambon Ralph, G. Pobric, E. Jefferies, Conceptual knowledge is underpinned by the temporal pole bilaterally: convergent evidence from rTMS. *Cereb Cortex* **19**, 832-838 (2009).

3. G. Pobric, E. Jefferies, M. A. Ralph, Anterior temporal lobes mediate semantic representation: mimicking semantic dementia by using rTMS in normal participants. *Proc Natl Acad Sci U S A* **104**, 20137-20141 (2007).

4. G. Pobric, M. A. Lambon Ralph, E. Jefferies, The role of the anterior temporal lobes in the comprehension of concrete and abstract words: rTMS evidence. *Cortex* **45**, 1104-1110 (2009).

5. J. Jung, S. R. Williams, F. Sanaei Nezhad, M. A. Lambon Ralph, GABA concentrations in the anterior temporal lobe predict human semantic processing. *Sci Rep* **7**, 15748 (2017).

6. R. J. Binney, K. V. Embleton, E. Jefferies, G. J. Parker, M. A. Ralph, The ventral and inferolateral aspects of the anterior temporal lobe are crucial in semantic memory: evidence from a novel direct comparison of distortion-corrected fMRI, rTMS, and semantic dementia. *Cereb Cortex* **20**, 2728-2738 (2010).

7. M. Visser, E. Jefferies, K. V. Embleton, M. A. Lambon Ralph, Both the middle temporal gyrus and the ventral anterior temporal area are crucial for multimodal semantic processing: distortion-corrected fMRI evidence for a double gradient of information convergence in the temporal lobes. *J Cogn Neurosci* **24**, 1766-1778 (2012).
